# Species-specific discrimination of bacterial biofilms using a ratiometric fluorescence sensor array and machine learning

**DOI:** 10.1039/d5sd00152h

**Published:** 2025-11-11

**Authors:** Ritika Gupta, Aayushi Laliwala, Elena Muldiiarova, Kenneth W. Bayles, Denis Svechkarev, Marat R. Sadykov, Aaron M. Mohs

**Affiliations:** a Department of Pharmaceutical Sciences, University of Nebraska Medical Center Omaha Nebraska 68198-6858 USA aaron.mohs@unmc.edu; b Department of Pathology, Microbiology and Immunology, University of Nebraska Medical Center Omaha Nebraska 68198-5900 USA msadykov@unmc.edu; c Department of Chemistry, University of Nebraska at Omaha Omaha Nebraska 68182-0109 USA dsvechkarev@unomaha.edu; d Fred and Pamela Buffet Cancer Center, University of Nebraska Medical Center Omaha Nebraska 68198-5900 USA; e Department of Biochemistry and Molecular Biology, University of Nebraska Medical Center Omaha Nebraska 68198-6858 USA

## Abstract

Biofilms are intricate bacterial communities encased in a self-produced extracellular matrix (ECM) of DNA, lipids, proteins, and polysaccharides. The diverse ECM composition across bacterial species significantly influences the progression of biofilm-associated infections, making precise identification crucial for effective treatment. Traditional methods such as biochemical assays, MALDI-TOF mass spectrometry, DNA sequencing and culturing provide valuable insights but have notable drawbacks, including time-consuming procedures, high costs, and the need for specialized equipment and trained personnel. These limitations hinder the rapid and widespread adoption of biofilm identification in clinical settings, underscoring the need for more streamlined, accurate, and accessible methods. In this study, we employed a paper-based ratiometric sensor array with fluorescent dyes (3-hydroxyflavone derivatives) pre-adsorbed onto paper microzone plates to identify bacterial biofilms. The fluorescence signals from the sensor upon interaction with biofilms were analyzed using linear discriminant analysis and different machine learning algorithms, including neural networks, support vector machines, and naïve Bayes classifiers. Our results show that the sensor array accurately distinguishes between biofilms of eight species with 97.5% classification accuracy. It effectively identifies individual bacteria at OD_600_ as low as 0.002 o.u. Additionally, using neural networks, the sensor array achieves more than 95% accuracy in distinguishing planktonic bacteria from biofilms and shows over 85% accuracy in identifying clinical bacterial species and biofilms. These findings highlight the sensor's potential for high-precision biofilm identification in laboratory and clinical settings, offering a valuable tool for advancing biofilm research and enhancing clinical diagnostics.

## Abstract 2

A paper-based sensor array comprised of four ratiometric fluorescent dyes to analyze bacterial biofilms. Using pattern recognition, it can identify biofilm-forming species and distinguish between planktonic cultures and biofilms with high accuracy.

## Graphical abstract

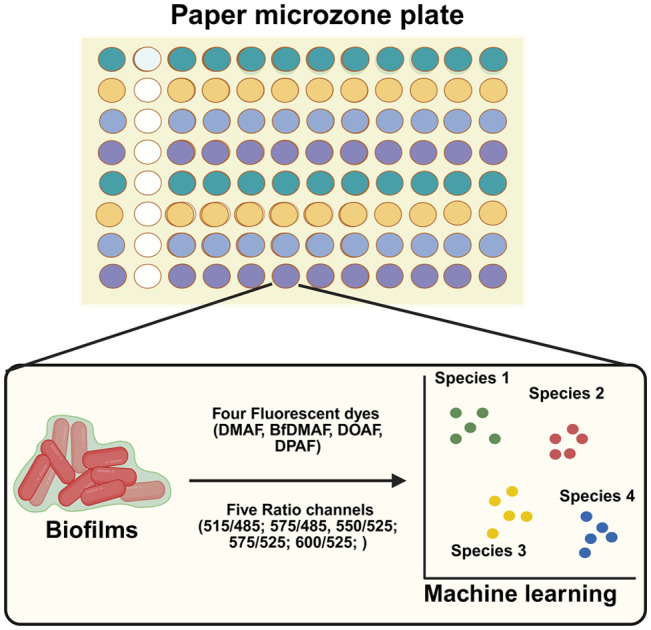

## Introduction

Bacterial biofilms, characterized by their ability to establish chronic infections and exhibit heightened resistance to antimicrobial treatments, pose a significant challenge in infectious disease medicine.^1–3^ These intricate communities of bacteria reside within a self-produced extracellular matrix (ECM), composed of proteins, extracellular DNA (eDNA), and polysaccharides, collectively referred to as extracellular polymeric substances (EPS).^2,4,5^ The biofilm ECM provides essential structural support and acts as a protective shield, effectively safeguarding the bacteria from host immune defenses and enhancing their resistance to eradication procedures and antimicrobial agents.^3,4,6–9^ These intrinsic ECM features largely contribute to the persistence of infections and microbial colonization associated with biofilms, underscoring their significant healthcare challenge.^8,9^ Concerns regarding bacterial biofilms in medical settings and antibiotic resistance have recently been highlighted by the World Health Organization (WHO), emphasizing the significance of developing innovative and sensitive strategies for identifying and characterizing biofilms.^10^ Early and accurate identification of bacterial biofilms is crucial for effectively managing and treating biofilm-associated infections, directly impacting patient outcomes and healthcare costs. Therefore, there is an urgent demand for developing novel drugs and therapeutic strategies to eradicate bacterial biofilms, establish effective surface treatment protocols, and impede bacterial adhesion and biofilm formation. Accurate identification allows targeted therapies, improved patient care, and a more effective approach to combat biofilm-associated infections.

Several techniques have been developed to detect and monitor bacterial biofilm dynamics. Traditional methods include culturing techniques, biochemical assays, MALDI-TOF, DNA sequencing and microscopic analysis, such as confocal laser scanning microscopy (CLSM) and scanning electron microscopy (SEM).^11^ While effective, these methods are often time-consuming and require extensive sample preparation. CLSM, for instance, provides detailed three-dimensional images of biofilms but it need fluorescent probes that may not bind uniformly to all biofilm components.^12^ SEM offers high-resolution images and is useful for understanding biofilm surface structures, but the sample preparation process is tedious and can introduce artifacts due to dehydration and coating requirements.^12^ More advanced techniques involve using biosensors to detect specific biofilm characteristics such as pH, oxygen levels, and ion concentrations.^13^ Technologies employed for biofilm detection include electrochemical sensors, optical sensors, and impedance spectroscopy.^14,15^ Electrochemical sensors can detect changes in biofilm activity by measuring electrical signals, while optical sensors, such as those using surface plasmon resonance, can monitor biofilm formation in real-time by detecting changes in light reflection.^16^ Impedance spectroscopy, a non-invasive electrochemical technique, measures the complex electrical impedance of biofilms across a range of frequencies, providing insights into biofilm formation, structure, and metabolic activity.^17^ Despite these advancements, there is still a pressing need for more rapid, accurate, and non-destructive methods for biofilm detection, especially in clinical settings where timely diagnosis is crucial.

Traditional sensors, which operate on a lock-and-key principle where a single, specific recognition element is designed to interact exclusively with the target analyte, often fall short of meeting these requirements for biofilm detection.^18^ This is because biofilms are complex, heterogeneous structures with varying compositions across species and environments, making them challenging to detect using conventional single-reporter sensing approaches. An array-based sensing approach has been developed to address this limitation, enabling the identification of various bacterial species by intrinsically non-specific cross-reactive reporters. This sensing approach comprises multiple recognition elements simultaneously and differentially interacting with the analyte.^18^ This interaction generates fingerprint patterns specific to the analytes of interest. These fingerprint patterns are used to identify and classify bacteria using pattern recognition analysis and other machine learning algorithms.^19,20^ Machine learning algorithms are categorized as supervised and unsupervised, in which supervised learning algorithms classify data based on the categories provided, whereas unsupervised learning classifies data based on clustering and similarities in patterns in unknown categories.^21^ Among the various algorithms used for bacterial sensing, commonly applied supervised learning algorithms include linear discriminant analysis (LDA), neural networks (NN), support vector machines (SVM), *k*-nearest neighbors (kNN), decision tree (DT), random forest (RF), naïve Bayes (NB). Unsupervised learning algorithms include hierarchical cluster analysis (HCA) and principal component analysis (PCA).^21,22^

Array-based sensors aided with machine learning algorithms offer rapid and accurate identification of unknown groups, thus improving the time of analysis for point-of-care tools.^22^ Derivatives of 3-hydroxyflavone have previously been reported as fluorescent sensors for identifying bacterial species.^23–25^ The four dyes employed in the current study possess different substituents at the same core, thus leading to different interactions with bacterial cell envelope components. The chemical structures of the four dyes are provided in Fig. S2. The primary mechanism of action involves van der Waals and hydrogen bonding interactions with cell envelope components, as well as spectral response variations depending on the polarity of the dye's microenvironment. The substituents are introduced to modulate dye interaction and binding; for instance, the long octyl chains in DOAF facilitate partitioning into hydrophobic regions of the bacterial cell envelope, while the phenyl groups in DPAF enhance π–π interactions with aromatic components of the membrane.^24^ As reported previously, these dyes undergo excited-state intramolecular proton transfer (ESIPT), producing two distinct emission bands. Moreover, they can participate in protolytic equilibria; under basic conditions or in the presence of strong hydrogen bond acceptors, 3-hydroxyflavones form anions or anion-like species with intermolecular proton transfer with fluorescence emission between the normal and tautomer bands.^24^ Consequently, these dyes do not exhibit a single emission band. We have established that these sensor dyes, immobilized on paper microzone plates, can differentiate bacterial species, identify their Gram status,^23^ and differentiate between drug-resistant and drug-sensitive *S. aureus* species.^26^ The paper plate–based sensor array has been demonstrated to be stable for up to 24 weeks, maintaining consistent fluorescence signal intensity when stored in the dark at room temperature.^23^ Paper-based sensing platforms have unique advantages as analytical tools: they can be designed for measuring absorbance, fluorescence, Raman scattering, and immobilize nanoparticles for bacterial species differentiation. In addition, paper-based platforms represent a significant advancement in point-of-care biofilm diagnostics. They offer simple to use, cost-effective, and highly accurate methods for bacteria detection and characterization, eliminating the need for complex instrumentation and extensive training.^27^

**Fig. 1:**
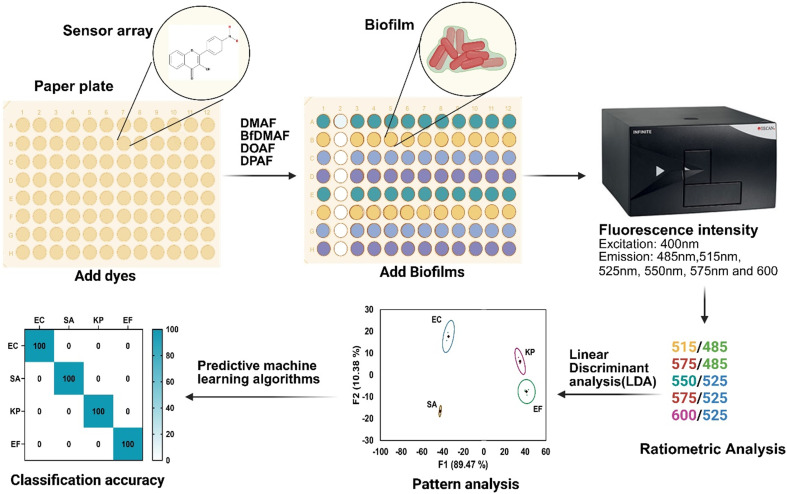
Schematic representation of machine learning-assisted biofilm identification: paper microzone plates immobilized with sensor array interact with biofilms, which generates unique responses. These data are analyzed using pattern recognition algorithms that differentiate individual species with improve classification accuracy (created using BioRender).

In this study, we investigate the potential of paper microzone plates pre-absorbed with an array of environment-sensitive fluorescent dyes and combined with machine learning algorithms to identify and differentiate bacterial biofilms (Fig. 1). We also assess the capability of the machine learning algorithms to classify bacterial species at the lowest concentrations of biofilm-forming bacteria. This study serves as a proof of concept, demonstrating that the paper-based sensor array, when combined with machine learning, can effectively differentiate and identify biofilms formed by different bacterial species, rather than merely detecting their presence. While clinicians already recognize the prevalence of biofilms in chronic and device-associated infections, the ability to distinguish between biofilm species is crucial for guiding diagnostic and therapeutic decisions. The results presented here establish the foundational step toward future clinical translation of this platform for pathogen identification in complex biological samples.

## Experimental section

### Materials

Polyisoprene-based negative photoresist-I (SC), xylenes histological grade, agarose, sodium chloride, human plasma, and glucose were purchased from Sigma-Aldrich (St. Louis, MO). Whatman paper grade 1 sheets were purchased from Cytiva Life Sciences (Marlborough, MA). Methanol HPLC grade was purchased from Millipore Sigma (Burlington, MA). 10× phosphate-buffered saline (PBS) was purchased from Mediatech, Inc. (Manassas, VA). Borosilicate glass plates were purchased from ThermoFisher Scientific (Waltham, MA). Tryptic soy broth and agar were purchased from BD Biosciences (Franklin Lakes, NJ). All aqueous solutions were prepared in deionized water. Four derivates of 3-hydroxyflavone (DMAF, BfDMAF, DOAF, DPAF) were used as sensor array and synthesized using the two-stage Algar–Flynn–Oyamada technique described in our previous article.^28^

### Fabrication of the paper-based sensor array

The fabrication of paper microzone plates was done using the method developed by Carrilho, *et al.* with modifications.^29^ The modified method was described in detail in the previously published article by Laliwala *et al.*^23^ Briefly, grade 1 Whatman filter paper (13 × 8.5 cm) was evenly coated with negative photoresist-I and dried for 1 hour. The coated plate was further aligned on a specially designed 96-well plate photomask and exposed for 15 s on both sides to UV radiation (315–400 nm) using a Sunray 600UV flood lamp (Uvitron International, Inc., West Springfield, MA). Further, the coated plates were washed in xylene for 5 min, followed by two washes in 100% methanol for 5 min each, and stored in a dark environment at room temperature. The fluorescent dyes were dissolved in ethanol to an OD_400_ = 2 (1 cm path length) for the detection assays and loaded (1 μl per well) on the fabricated paper plates.

### Strains, growth conditions, and static biofilm assay

Bacterial species used in the study were from our lab collection and included four Gram-negative bacteria: *Escherichia coli* DH5α, *Proteus vulgaris*, *Klebsiella pneumoniae*, and *Acinetobacter baumannii*, as well as four Gram-positive bacteria: *Staphylococcus aureus* UAMS1, *Bacillus subtilis* 168, *Enterococcus faecalis*, and *Staphylococcus epidermidis* 1457. Planktonic bacterial cultures and biofilms were prepared as previously reported.^30^ Briefly, bacteria were grown in tryptic soy broth (TSB) medium (BD Biosciences) supplemented with 0.25% glucose. Bacterial cells were collected from overnight cultures after 15 h of growth (37 °C shaking incubator at 250 rpm) by centrifugation at 15 100*g*, washed twice with 1× PBS and resuspended in 200 μL of fresh PBS to OD_600_ = 20 optical units (o.u.). Biofilms were grown on sterile polystyrene 96-well flat-bottom microtiter plates (Corning, Inc.), pre-coated with 200 μL of 20% human plasma in carbonate buffer, and incubated overnight at 4 °C. Overnight-grown bacterial cultures were diluted in fresh TSB, supplemented with 0.5% glucose and 3% sodium chloride, to OD_600_ = 0.05 o.u. 200 μL of cultures were used to inoculate wells of pre-coated microtiter plates, and biofilms were grown statically for 72 h at 37 °C. The growth media was removed, biofilms were disrupted by scraping using micropipettes, washed with 1× PBS, and concentrated in 200 μL of PBS to OD_600_ = 20 o.u. (Fig. S1). The culture conditions for Gram-positive and Gram-negative strains were the same throughout the study. Planktonic and biofilm samples (5 μL per well, 10 replicates per sample) were loaded onto the paper microzone plates with pre-adsorbed sensor dyes. The samples were allowed to interact with the sensor dyes for 1 h. For the experiments, we utilized 4 sensor dyes × 5 ratio channels × 10 replicates, for each species. Fluorescence intensity was recorded using a Tecan 200 spectrofluorometric plate reader. Five independent measurements at *λ*_em_ = 485, 515, 525, 550, 575, and 600 nm (*λ*_ex_ = 400 nm) were collected for each well. For the limit of detection experiment, the cultures were diluted from 20 o.u. to 2, 0.2, 0.02 and 0.002 o.u. Different bacterial and biofilm concentrations were used to differentiate planktonic bacterial species from their respective biofilms. Clinical isolates were obtained from the Clinical Microbiology Laboratory at Nebraska Medicine, cultured, and treated as described above.

### Data processing and analysis

Six independent fluorescence intensities at emission 485, 515, 525, 550, 575, and 600 nm were recorded using a Tecan Infinite 200 spectrofluorometric plate reader. These fluorescence intensities were used to calculate ratios at five combinations: 515/485, 575/485, 550/525, 575/525, and 600/525. The five intensity ratios for all four dyes were used as features (a total of 20 features for every experimental point). The calculated data from all dyes (DMAF, BfDMAF, DOAF, and DPAF) were analyzed using different classification algorithms such as LDA, kNN, NN, SVM, NB and RF. For linear discriminant analysis, all data point were used for dimensionality reduction and visualization of the differentiation performance using canonical score plots. For other supervised learning methods, the dataset was randomly split into 75% used for training and 25% used for validation while preserving equal representation of all classes in both training and validation subsets. The classification algorithms were used with their default settings and no further optimization was performed. 2-Dimensional (2D) canonical plots were obtained using the XLSTAT software package (Addinsoft corporation, version 2023.3.0.1415).^31^ Other classification algorithms were applied using Orange Data Mining software.^32^

## Results and discussion

### Discrimination of bacterial biofilms

**Fig. 2:**
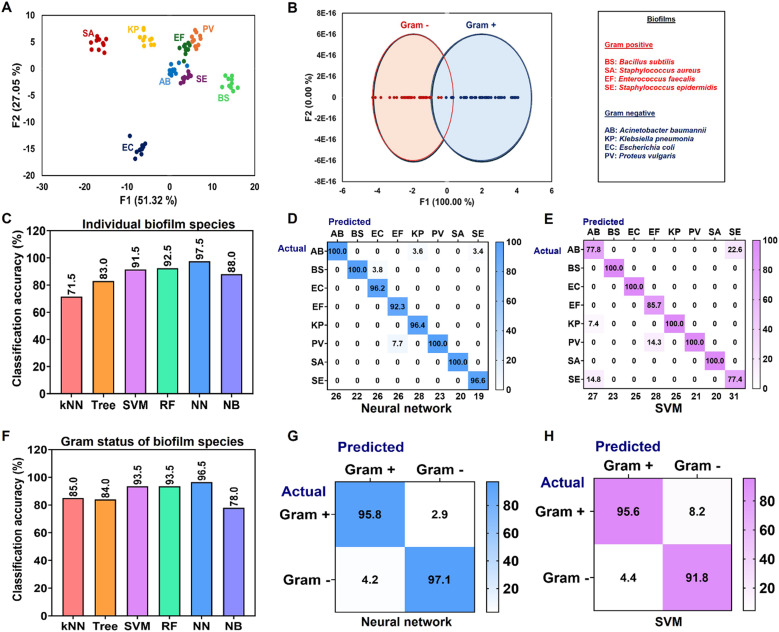
Identification of bacterial biofilms. (A) 2D canonical plot obtained by LDA for differentiation of biofilms formed by different species; (B) 2D canonical plot obtained by LDA for differentiation of biofilms based on Gram status; (C) percentage accuracy in classifying individual biofilm-forming bacterial species; (D) confusion matrix for NN classification of individual biofilm-forming species; (E) confusion matrix for SVM classification of individual biofilm-forming species; (F) percentage accuracy in categorizing biofilm species as Gram-positive or Gram-negative; (G) confusion matrix for NN classification of Gram-positive and Gram-negative biofilm-forming species; (H) confusion matrix for SVM classification of Gram-positive and Gram-negative biofilm-forming species.

The paper-based sensor array was employed to distinguish species-specific bacterial biofilms, including four Gram-positive species [*S. aureus* (SA), *E. faecalis* (EF), *S. epidermidis* (SE), and *B. subtilis* (BS)] and four Gram-negative species [*E. coli* (EC), *A. baumannii* (AB), *P. vulgaris* (PV), and *K. pneumoniae* (KP)]. The linear discriminant canonical plot showed that our sensor can differentiate between biofilms from eight bacterial species (Fig. 2A) and categorize them by their Gram status (Fig. 2B). Some clusters on the LDA score plot are quite close to each other, thus prompting the use of more advanced classification algorithms for improved differentiation. The sensor array signals data were subjected to comprehensive analysis using pattern recognition algorithms including KNN, NN, SVM, RF, DT, and NB. This approach aimed to differentiate bacterial biofilms as individual species and according to their Gram status. To ensure robust validation, the dataset was randomly divided into two subsets: 75% for training and 25% for testing. Among the algorithms tested, NN demonstrated superior performance. NN successfully discriminated biofilm-forming bacteria into individual species with an accuracy of 97.5% (Fig. 2C) and categorized them into broader groups (Gram-positive and Gram-negative) with 96.5% accuracy (Fig. 2F). As shown in Fig. 2D and E, the confusion matrices for NN and SVM demonstrate that more than 95% of instances were correctly classified. For example, in Fig. 2D, 96.2% of the 26 data points were accurately predicted to belong to *E. coli*. Similar trends were observed for other species. Fig. 2G and H show that NN and SVM correctly distinguished species based on their Gram status with an accuracy exceeding 90%. The confusion matrices for individual strains using NN and SVM are provided in Tables S1 and S2, respectively, while those for Gram status classification are presented in Tables S3 and S4.

Gram-positive bacteria have a thick, single-layered peptidoglycan cell wall (20 to 80 nm thick) without an outer membrane or lipopolysaccharide, whereas Gram-negative bacteria have a much thinner peptidoglycan layer (∼2 to 3 nm), which is located between an inner cytoplasmic membrane and an outer membrane that contains lipopolysaccharide.^33–35^ Polysaccharides play an important role in biofilm formation, and the distinct composition of these molecules in Gram-positive and Gram-negative bacteria contributes significantly to differences in their respective biofilm matrix components.^36^ Additionally, Gram-positive and Gram-negative bacteria produce different quorum-sensing molecules – autoinducing peptides (AIPs) and acyl-homoserine lactones (AHLs), respectively – which influence biofilm formation.^37^ These variations result in distinct polarities of bacterial and biofilm matrix components, ultimately modulating the interaction between sensor dyes and biofilms.^26^ Despite these differences, both Gram-positive and Gram-negative biofilms share some similar matrix components, which can make complete differentiation challenging.^34^ The complex interplay between shared and distinct features in biofilm composition highlights the need for sophisticated detection methods that can accurately distinguish between different bacterial species in biofilm communities.

### Determination of sensing limit using different biofilm loads

**Fig. 3:**
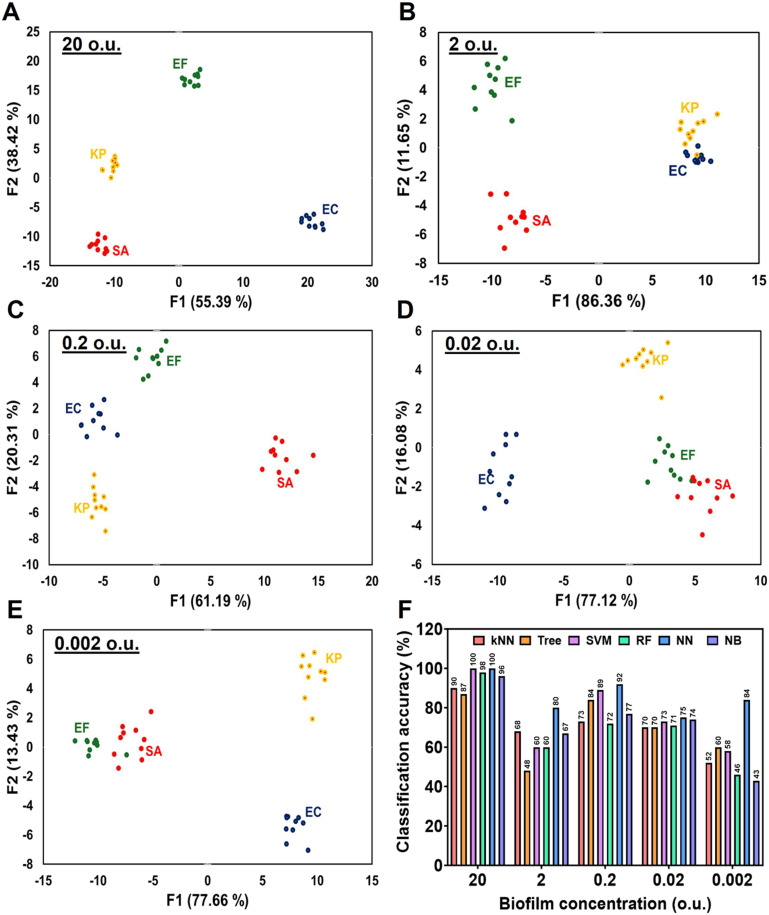
2D canonical plots using LDA for different biofilm loads. (A) 20 o.u.; (B) 2 o.u., (C) 0.2 o.u., (D) 0.02 o.u. and (E) 0.002 o.u. (F) Classification accuracy of SA, EC, KP, and EF differentiation at various biofilm loads using various machine learning algorithms.

The sensitivity of the sensor array for biofilm detection was evaluated using various biofilm concentrations. Biofilms were serially diluted to 20, 2, 0.2, 0.02, and 0.002 o.u. at 600 nm, and 5 μL of each dilution was applied to paper microzone plates pre-loaded with fluorescent sensor dyes. Fluorescence intensity was measured as previously described and analyzed using machine learning algorithms.^23,24,26^Fig. 3(A–E) displays 2D canonical plots obtained by LDA for all biofilm concentrations. Notably, Fig. 3E demonstrates the sensor array's ability to differentiate biofilms at the lowest concentration of 0.002 o.u. Classification accuracy was further analyzed across concentration levels, as illustrated in Fig. 3F. At the highest concentration (20 o.u.) SVM, NB, and NN algorithms achieved classification accuracies of 100%, 96%, and 100%, respectively. However, at 0.002 o.u., accuracy decreased to less or equal to 60% for most algorithms, except for NN, which shows 84% accuracy. Based on these results, we concluded that 0.002 o.u. is the lowest biofilm concentration that can be reliably identified using LDA and other machine learning algorithms, with over 50% classification accuracy. Therefore, this concentration was considered the sensor's limit for identifying of bacterial biofilms above 50% accuracy. Additional analyses of fluorescence intensity and ratiometric dynamics are presented in Fig. S3 and S4, respectively. These figures demonstrate that fluorescence intensity remains stable across all ratios, regardless of biofilm concentration. These findings highlight the potential of this paper-based sensor array for identifying and differentiating biofilms across a range of concentrations, offering a promising tool for biofilm detection and characterization.

### Differentiation of planktonic bacteria and biofilms at various loads

**Fig. 4:**
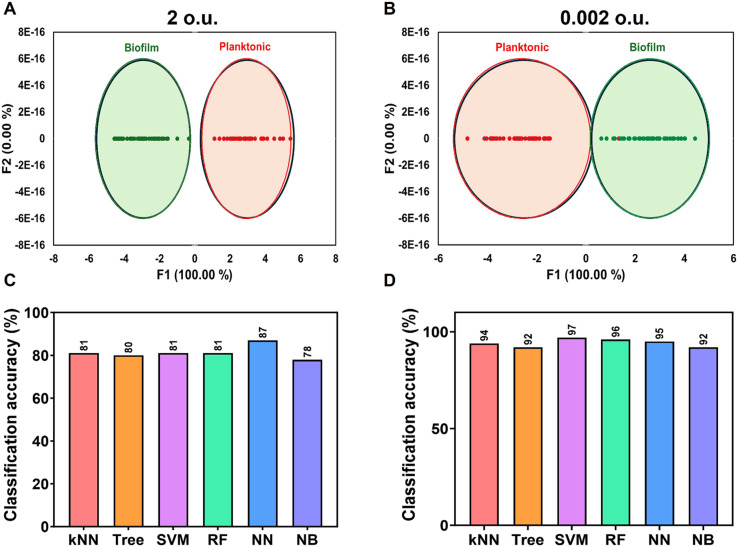
Differentiation of planktonic bacteria and biofilms. (A) 2D canonical plot of planktonic bacteria and biofilms at 2 o.u. (B) 2D canonical plot of planktonic bacteria and biofilms at 0.002 o.u. (C) Classification accuracy at 2 o.u.; (D) classification accuracy at 0.002 o.u.

The sensor array was evaluated for its ability to differentiate biofilms and planktonic bacteria of the same species at two different concentrations. Interestingly, the differentiation was more effective at 0.002 o.u. compared to 2 o.u. (Fig. 4A and B). The sensor successfully distinguished biofilms from their respective planktonic bacteria. Further analysis using machine learning algorithms revealed more than 95% classification accuracy at 0.002 o.u. At 2 o.u., the accuracy decreased slightly to 81% and 87% using RF and NB algorithms, respectively, as shown in Fig. 4C and D. The concentration-dependent performance of the sensor array can be explained by how varying biofilm loads influence the interaction between sensor dyes and biofilm components. At higher optical densities, the increased presence of biofilm material, including both ECM and bacterial cells, may lead to saturation effects, where the sensor dyes become overwhelmed, limiting their ability to distinguish subtle differences between biofilm and planktonic samples. Additionally, the denser biofilm could impede dye diffusion, reducing interaction with deeper biofilm structures. The higher concentration of biofilm ECM may also increase background fluorescence, potentially masking the specific signals used for differentiation. Conversely, at lower optical units, the reduced biofilm load allows for more efficient dye penetration, enabling the sensor dyes to interact more thoroughly with ECM and bacterial cells. This lower concentration also enhances signal clarity by reducing background interference, leading to more accurate detection of specific interactions. Furthermore, the simplified biofilm environment at lower loads accentuates differences between biofilm and planktonic bacteria, making them easier to distinguish.

### Identification of clinical isolates in planktonic and biofilm cultures

**Fig. 5:**
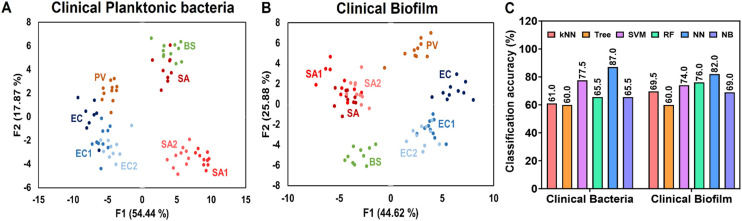
Identification of clinical isolates in planktonic and biofilm cultures. (A) 2D canonical plot of planktonic bacterial cultures from clinical isolates and laboratory strains. (B) 2D canonical plot of biofilms formed by clinical isolates and laboratory strains; (C) classification accuracy percentages for identification of clinical isolates in planktonic cultures and biofilms.

The sensor array's clinical applicability to differentiate between bacterial species as planktonic and biofilm-forming bacteria was further evaluated using both laboratory strains and clinical isolates. Fig. 5A illustrates the clustering patterns of planktonic bacteria, while Fig. 5B shows the results for biofilms. In both cases, distinct groupings provide insights into the sensor's discriminatory capabilities. In Fig. 5A, planktonic *E. coli* clinical isolates (EC1 and EC2) clustered closely with the laboratory *E. coli* (EC) strain, showing a good separation from *P. vulgaris* (PV). In Fig. 5B, representing biofilms, a similar trend was observed for *E. coli*, with EC1 and EC2 biofilms clustering closer to the laboratory EC biofilm than to PV. Similarly, the clinical biofilm *S. aureus* isolates (SA1 and SA2) grouped closer to the laboratory *S. aureus* (SA) strain than to *B. subtilis* (BS). The clear separation between *E. coli* and *P. vulgaris* (Gram-negative) and *S. aureus* and *B. subtilis* (Gram-positive) highlights the differences in cell envelope composition and structure between these bacterial groups. In both cases, clusters of Gram-positive bacteria localize on one side of the LDA score plot, whereas clusters of Gram-negative bacteria are found on the opposite side: a behavior first observed and reported in our earlier work.^24^ These patterns reflect strong species-specific characteristics that remain consistent between laboratory and clinical strains. Subtle differences in positioning between clinical isolates and laboratory strains likely arise from variations in surface proteins and/or extracellular matrix composition. Interestingly, the Gram-positive planktonic bacteria showed a different pattern: the laboratory SA strain was positioned between the clinical *S. aureus* isolates (SA1 and SA2) and *B. subtilis*, and closer to *B. subtilis*. Several factors might contribute to these clustering patterns. The tight grouping of clinical isolates with their corresponding laboratory strains underscores the preservation of species-specific characteristics, detectable by the sensor array. The intermediate positioning of the laboratory *S. aureus* strain between clinical isolates and *B. subtilis* may reflect adaptations acquired during extended cultivation under controlled laboratory conditions, possibly altering surface proteins. Finally, the clustering patterns are influenced by specific interactions between sensor array components and bacterial surface molecules or ECM, leading to consistent responses within species but variations between Gram-positive and Gram-negative bacteria. Using NN, the sensor array demonstrated high classification accuracy for both planktonic bacteria and biofilms from clinical isolates. Specifically, NN achieved an accuracy of 87% for clinical planktonic bacteria and 82% for clinical biofilms, as illustrated in Fig. 5C. These results demonstrate the sensor array's capacity to detect subtle differences between bacterial species and strains, effectively distinguishing between laboratory and clinical isolates. This capability is especially pronounced in biofilms, where the complex extracellular matrix introduces additional factors for differentiation. Overall, these findings underscore the potential of the sensor array as a powerful tool for bacterial identification in both research and clinical settings. Its ability to discriminate between closely related strains and detect biofilm composition differences could significantly enhance diagnostic applications, particularly when rapid and accurate identification of bacterial species and their growth states is critical.

The sensor's high accuracy in identifying clinical strains, which share similarities with laboratory strains, further highlights its clinical relevance. These results underscore the sensor array's robust ability to distinguish between different bacterial species in their planktonic and biofilm modes of growth in clinical samples. The sensor array's design, which enables interactions with both the EPS matrix and bacterial cell envelope components, contributes significantly to its ability to differentiate strains based on their unique molecular signatures. This feature allows the sensor to detect subtle differences between species and strains. Furthermore, the sensor array's capacity to distinguish between planktonic and biofilm modes of bacterial existence adds another layer of diagnostic value. This ability is particularly crucial in clinical settings, where the differentiation between these two bacterial lifestyles can significantly impact treatment strategies. The sensor array's demonstrated proficiency in identifying both biofilms and planktonic bacteria positions it as a promising tool for addressing the challenges associated with biofilm diagnosis in clinical environments. Its high accuracy and versatility make it a valuable asset for rapid and precise bacterial identification, potentially improving diagnostic processes and informing more targeted treatment approaches. The ability to distinguish between different bacterial species, their planktonic and biofilm forms, underscores the sensor array's potential to significantly enhance current diagnostic capabilities in clinical microbiology.

## Conclusions

Accurate and reliable identification of biofilms formed by various bacterial pathogens is crucial for addressing the challenges of biofilm-associated infections in medical settings. These complex microbial communities, encased in a self-produced ECM, significantly contribute to antibiotic resistance and persistent infections. Therefore, paper-based ratiometric sensor array utilizing fluorescent sensor dyes was developed for effectively identifying various bacterial biofilms and distinguishing them from planktonic bacteria. This sensor shows clinical applicability by differentiating between Gram-positive and Gram-negative groups of bacterial biofilms. Integration of machine learning algorithms improves the sensing accuracy to 97.5% in distinguishing biofilms of four Gram-negative and four Gram-positive bacterial species, and demonstrates sensitivity at low concentrations of 0.002 o.u. While our sensor successfully differentiates single-species biofilms, there are certain limitations. The dyes interact non-specifically with ECM components of bacterial biofilms, which allows for analysis of a wide range of targets. At the same time, the use of LDA and machine learning algorithms for classification limits the applicability of conventional calibration methods. Overall, this sensor shows promising proof of concept and, as a point-of-care diagnostic tool by enabling rapid analysis, reducing time and costs compared to conventional methods. Its ability to distinguish bacterial biofilms addresses critical needs in clinical practice and research, supporting more effective treatment strategies and antimicrobial stewardship efforts. As we continue to refine this technology, we anticipate it will significantly improve the understanding and management of biofilm-related infections by incorporating various combinations of dual- and mixed-species biofilms, as well as additional species and strains, into the training datasets.

## Author contributions

RG: data curation, formal analysis, investigation, methodology, software, writing – original draft, writing – review & editing; AL – data curation, formal analysis, investigation, methodology, software, writing original draft, writing – review & editing; EM – investigation, methodology, editing; KWB – funding acquisition, resources, project administration; DS: conceptualization, methodology, investigation, validation, writing – review & editing; MRS – conceptualization, data curation, methodology, investigation, funding acquisition, supervision, validation, visualization, writing – original draft, writing-review & editing; AMM – conceptualization, funding acquisition, investigation, project administration, resource, supervision, validation, visualization, writing – review & editing.

## Conflicts of interest

The authors declare no conflict of interest.

## Supplementary Material

SD-005-D5SD00152H-s001

## Data Availability

The data supporting this article have been included as part of the supplementary information (SI). Supplementary information: schematic for biofilm sample preparation, chemical structures of the dyes used, additional experimental data including validation data for neural network and support vector machines for classifying individual Gram-positive and Gram-negative laboratory strains, validation data for for neural network and support vector machines for identification of Gram-positive and Gram-negative categorically, fluorescence intensity analysis of sensor dyes interaction with Gram-positive and Gram-negative strains at 550 nm, ratiometric analysis of DMAF interaction with Gram-positive and Gram-negative strains at five channels. See DOI: https://doi.org/10.1039/d5sd00152h.
