# Supplementary material for: Species-specific discrimination of bacterial biofilms using a ratiometric fluorescence sensor array and machine learning
Source: Sens Diagn. 2025 Nov 11;5(2):232–41. doi: 10.1039/d5sd00152h (PMC12658472; doi:10.1039/d5sd00152h)
Supplement: SD-005-D5SD00152H-s001 [file SD-005-D5SD00152H-s001.pdf]

Supplementary Information (SI) for Sensors & Diagnostics.
This journal is © The Royal Society of Chemistry 2025

Species-Specific Discrimination of Bacterial Biofilms Using a Ratiometric Fluorescence Sensor Array and Machine Learning

Ritika Gupta[a#], Aayushi Laliwala[a#1], Elena Muldiiarova[b], Kenneth W. Bayles[b], Denis Svechkarev[c*], Marat R. Sadykov[b*], Aaron M. Mohs[a,d,e*]

[a]Department of Pharmaceutical Sciences, University of Nebraska Medical Center, Omaha, Nebraska 68198-6858, United States

[b]Department of Pathology, Microbiology and Immunology, University of Nebraska Medical Center, Omaha, Nebraska 68198-5900, United States

[c]Department of Chemistry, University of Nebraska at Omaha, Omaha, Nebraska, 68182-0109, United States

[d]Fred and Pamela Buffet Cancer Center, University of Nebraska Medical Center, Omaha, Nebraska 68198-5900, United States

[e]Department of Biochemistry and Molecular Biology, University of Nebraska Medical Center, Omaha, Nebraska 68198-6858, United States

[#]RG and AL contributed equally to this work.

To Whom Correspondence Should be Addressed:

Aaron M. Mohs, Ph.D.
Department of Pharmaceutical Sciences,
University of Nebraska Medical Center,
Omaha, Nebraska 68198-6858, United States
Email: aaron.mohs@unmc.edu

Marat R. Sadykov, Ph.D.
Department of Pathology, Microbiology and Immunology,
University of Nebraska Medical Center,
Omaha, Nebraska 68198-6858, United States
Email: msadykov@unmc.edu

Denis Svechkarev, Ph.D.
Department of Chemistry,
University of Nebraska at Omaha,
Omaha NE, United States
Email: dsvechkarev@unomaha.edu

[1]Present address for AL: Department of Radiology, Case Western Reserve University, Cleveland, Ohio 44106, United States

Table of Contents

Figure S1…………………………………………………………………………Page 3

Figure S2…………………………………………………………………………Page 3

Table S1………………………………………………………………………… Page 4

Table S2………………………………………………………………………… Page 4

Table S3………………………………………………………………………… Page 5

Table S4………………………………………………………………………… Page 5

Figure S3……………………………………………………………………….. Page 6

Figure S4……………………………………………………………………….. Page 7

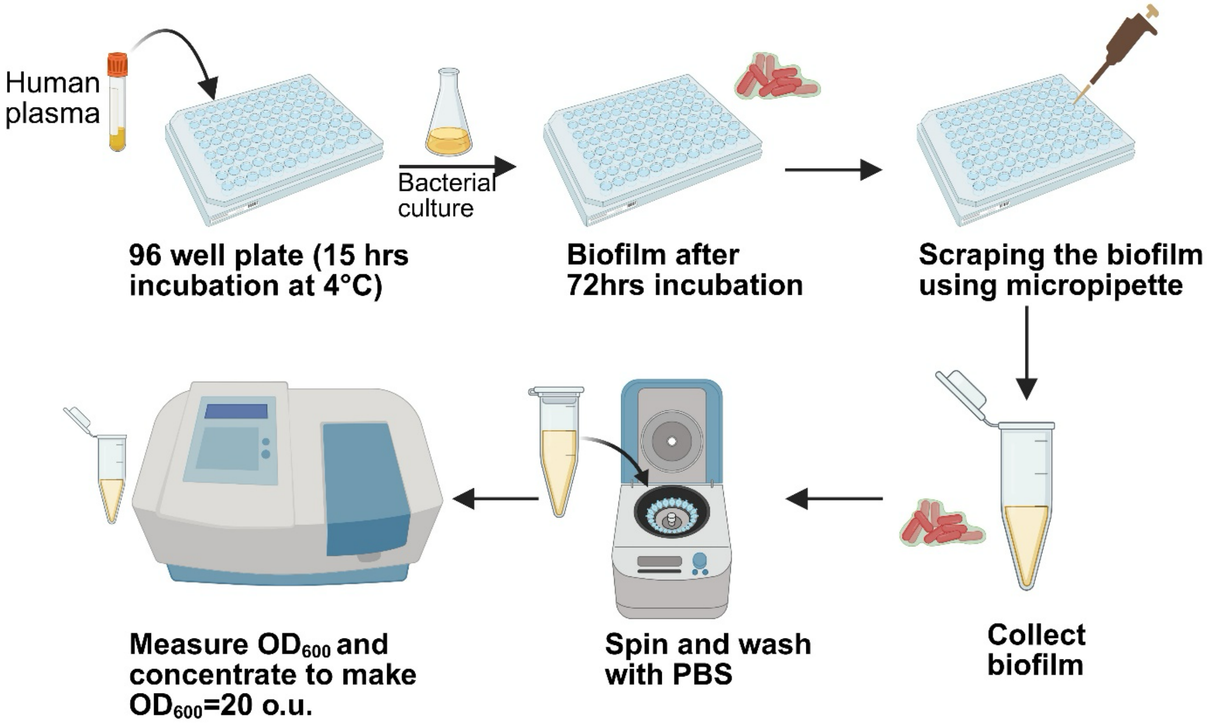

Figure S1: Biofilm sample preparation (Created using Biorender)

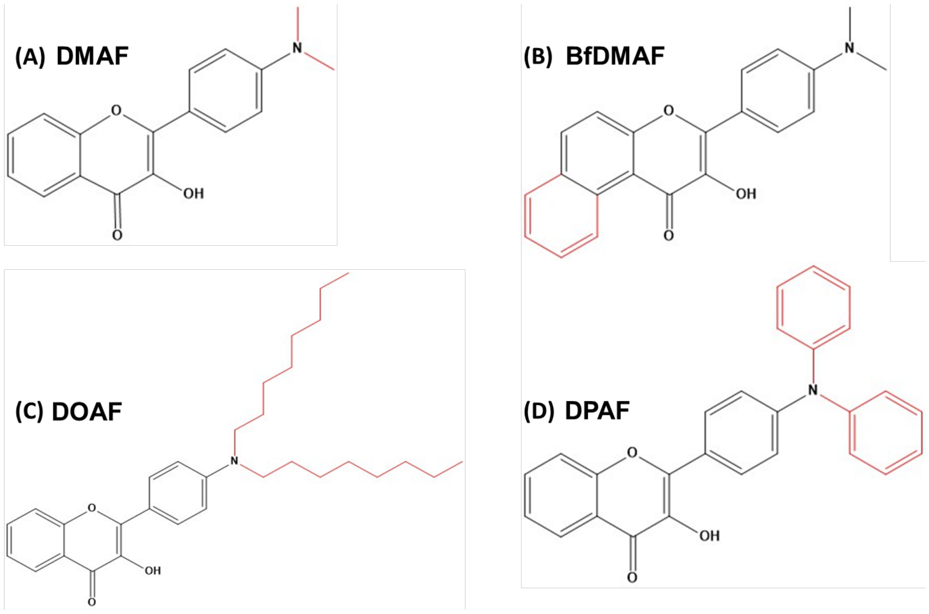

Figure S2: Chemical structure of the ratiometric dyes used in the study

Table S1. Validation results for neural networks classification of individual Gram-positive and Gram-negative laboratory species.

	Strains	Correctly identified	Present in training set	Classification accuracy (%)
Gram positive species	SA	20	20	100
	BS	22	22	100
	EF	24	26	92.30
	SE	28	29	96.5
Gram negative species	EC	25	26	96.15
	KP	27	28	96.4
	PV	23	23	100
	AB	26	26	100
	Total	195	200	97.5

Table S2. Validation for support vector machines classification of individual Gram-positive and Gram-negative laboratory species.

	Strains	Correctly identified	Present in training set	Classification accuracy (%)
Gram positive species	SA	20	20	100
	BS	23	23	100
	EF	24	28	85.7
	SE	224	31	77.4
Gram negative species	EC	25	25	100
	KP	25	25	100
	PV	21	21	100
	AB	21	27	77.7
	Total	183	200	91.5

Table S3. Validation results for neural network identification of Gram-positive and Gram-negative bacteria categorically.

Strains	Correctly identified	Present in training set	Classification accuracy (%)
**Gram-positive**	92	96	95.8
**Gram-negative**	101	104	97.1
**Total**	193	200	96.5

Table S4. Validation results for support vector machines identifications of Gram-positive and Gram-negative bacteria categorically.

Strains	Correctly identified	Present in training set	Classification accuracy (%)
**Gram-positive**	86	90	95.5
**Gram-negative**	101	110	91.8
**Total**	187	200	93.5

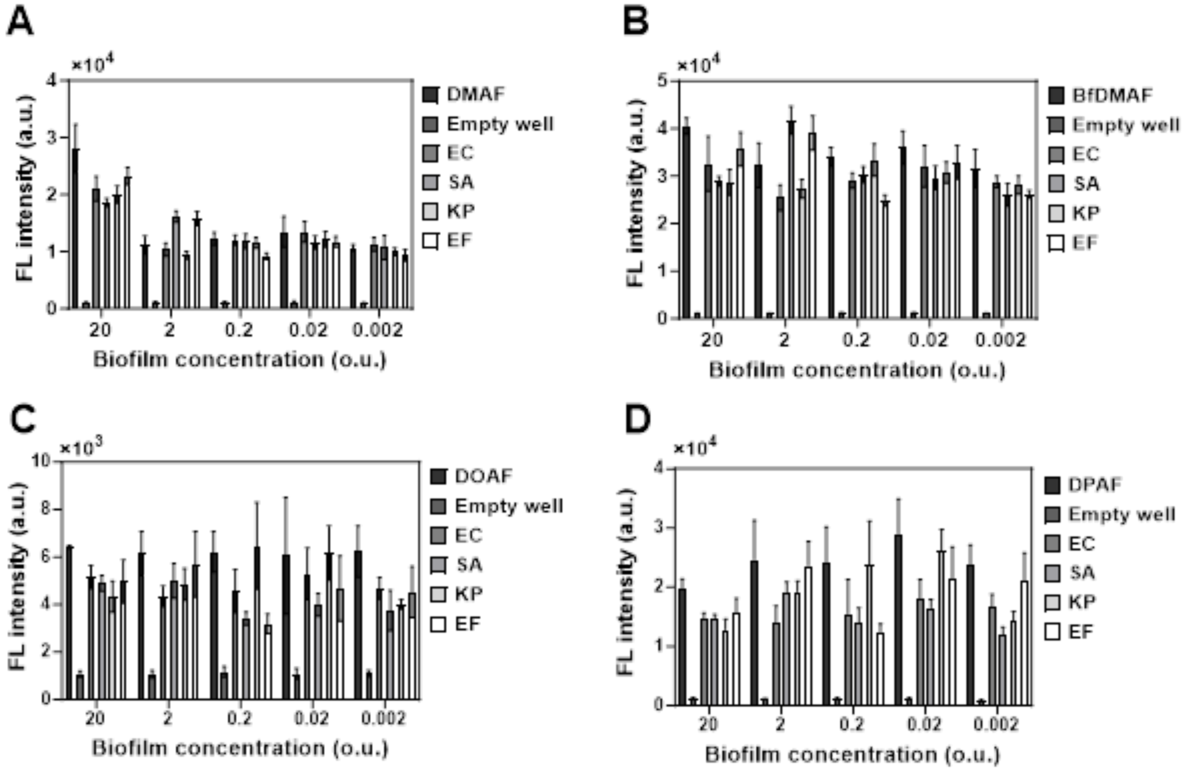

Figure S3. Fluorescence intensity analysis of sensor dye interactions with Gram-positive and Gram-negative bacteria at 550 nm (λ_{ex} = 400 nm) and various biofilms loads. (A) DMAF, (B) BfDMAF, (C) DOAF, and (D) DPAF.

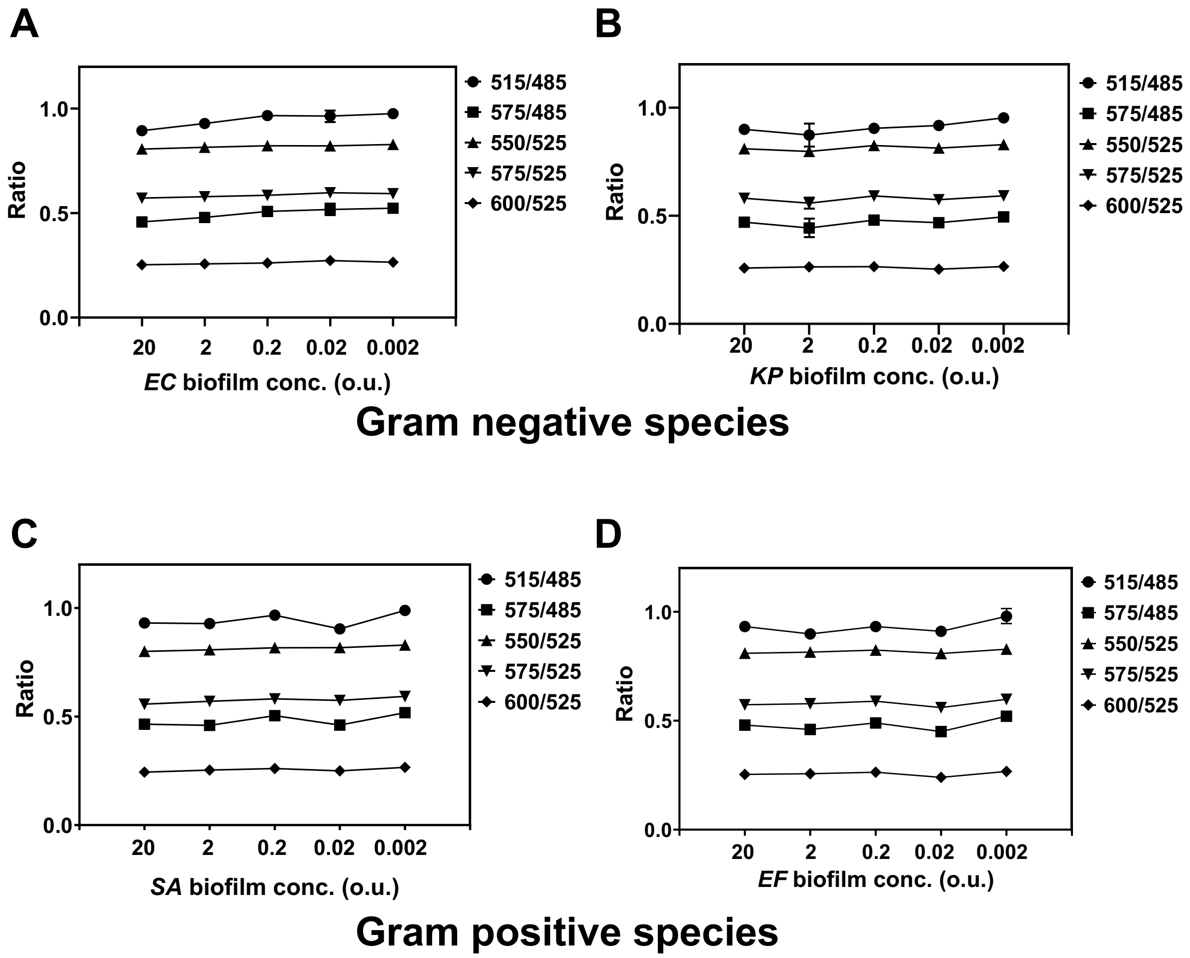

Figure S4. Ratiometric analysis of DMAF interaction with Gram-negative (A) EC, (B) KP, and Gram-positive (C) SA (D) EF bacteria at five channels (λ_{ex} = 400 nm).
